# The Influence of Physiological Aging and Atrophy on Brain Viscoelastic Properties in Humans

**DOI:** 10.1371/journal.pone.0023451

**Published:** 2011-09-12

**Authors:** Ingolf Sack, Kaspar-Josche Streitberger, Dagmar Krefting, Friedemann Paul, Jürgen Braun

**Affiliations:** 1 Department of Radiology, Charité-Universitätsmedizin Berlin, Campus Mitte, Berlin, Germany; 2 Institute of Medical Informatics, Charité-Universitätsmedizin Berlin, Campus Benjamin Franklin, Berlin, Germany; 3 NeuroCure Clinical Research Center, Charité-Universitätsmedizin Berlin, Campus Mitte, Berlin, Germany; 4 Experimental and Clinical Research Center, Charité-Universitätsmedizin Berlin and Max Delbrück Center for Molecular Medicine, Berlin, Germany; University of California, San Francisco, United States of America

## Abstract

Physiological aging of the brain is accompanied by ubiquitous degeneration of neurons and oligodendrocytes. An alteration of the cellular matrix of an organ impacts its macroscopic viscoelastic properties which can be detected by magnetic resonance elastography (MRE) – to date the only method for measuring brain mechanical parameters without intervention. However, the wave patterns detected by MRE are affected by atrophic changes in brain geometry occurring in an individual's life span. Moreover, regional variability in MRE-detected age effects is expected corresponding to the regional variation in atrophy. Therefore, the sensitivity of brain MRE to brain volume and aging was investigated in 66 healthy volunteers aged 18–72. A linear decline in whole-brain elasticity was observed (−0.75%/year, R-square = 0.59, p<0.001); the rate is three times that determined by volume measurements (−0.23%/year, R-square = 0.4, p<0.001). The highest decline in elasticity (−0.92%/year, R-square = 0.43, p<0.001) was observed in a region of interest placed in the frontal lobe with minimal age-related shrinkage (−0.1%, R-square = 0.06, p = 0.043). Our results suggest that cerebral MRE can measure geometry-independent viscoelastic parameters related to intrinsic tissue structure and altered by age.

## Introduction

Aging is an inevitable aspect of life. The human brain, too, is destined to undergo this process with mature brain tissue becoming progressively disorganized and degraded with age, indicated by a progressive loss of neurons and oligodendrocytes in the course of life [1]. This age-related neurodegeneration is likely to alter the cellular matrix of the brain and thus to have an impact on its viscoelastic properties such as softness, stiffness, and mechanical friction [2]. Brain atrophy occurring as a consequence of physiological aging as well as pathological conditions has been reliably quantified by conventional brain magnetic resonance imaging (MRI). However, the capability of conventional MRI to detect changes in brain viscoelastic properties is limited. Against this background, brain magnetic resonance elastography (MRE) [3] was developed as a novel method for detecting changes in cerebral viscoelasticity by combining MRI with acoustic waves [4], [5], [6], [7], [8], [9], [10]. Recently, we have shown that brain MRE can provide reproducible information on brain elasticity in healthy volunteers [11], [12] and subjects with neurological conditions such as multiple sclerosis [13] and hydrocephalus [14]. In particular, we demonstrated that stiffness of the adult brain is continuously decreasing with age [2]. However, recovery of elastic moduli from MRE wave images is based on the solution of the inverse problem of propagating shear waves, which is mathematically ill-posed and thus error prone [15], [16]. Hence, changing geometrical boundary conditions as given by brain atrophy in the course of aging may confound MRE-derived elasticity parameters. Therefore, we performed a cross-sectional exploratory study to investigate the sensitivity of brain MRE to physiological aging and variations in brain volume possibly influencing geometrical boundary conditions in wave inversion.

## Methods

### Sample

This study was approved by the ethics committee of the Charité Berlin (directives EA1/006/07 and EA1/182/07) and written informed consent was obtained from all participants. Sixty-six volunteers without overt neurological or psychiatric conditions were included in this study (mean age 45.92 years, standard deviation [SD] 16.21 years, age range 18 to 72 years; 31 men, mean age 42.58 years, SD 16.77 years, age range 20 to 72 years; 35 women, mean age 48.89 years, SD 15.33 years, age range 18 to 72 years).

### Data acquisition

**Figure 1:**
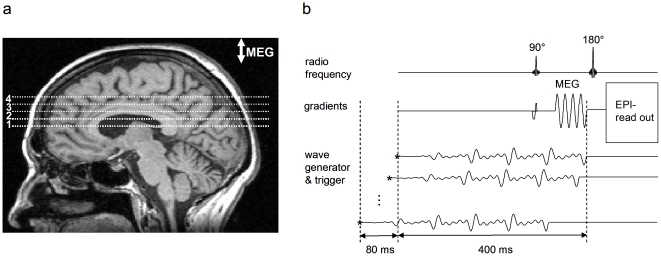
MRE experimental setup. **a:** Typical positions of four adjacent image slices used for multifrequency MRE (slice thickness 6 mm). The image slices were angulated around the left-right axis to compensate for neck flexion, yielding slices parallel to genu and splenium of the corpus callosum. **b:** Sketch of the imaging sequence. A spin-echo single-shot echo-planar imaging (EPI) sequence was sensitized to motion by a sinusoidal motion encoding gradient (MEG) comprising four cycles of 60 Hz sinusoids. MEG direction was through-plane. The wave generator was triggered by the sequence at the time points demarcated by the asterisks. The waveform was composed by four harmonic frequencies from 25 to 62.5 Hz. The wave trigger was shifted consecutively 32 times relative to the MEG in order to capture the propagation of the waves through the head.

Experiments were run on a standard 1.5 T clinical MRI scanner (Siemens, Erlangen, Germany). For MRI volumetry a magnetized prepared rapid gradient echo (MPRAGE) sequence (TR/TE  =  2110/4.4 milliseconds, TI 1100 ms, flip angle 15°, resolution 1 mm^3^) was used for acquiring three-dimensional brain data. For MRE four adjacent image slices were selected based on MRI volumetry data. The image slices were positioned in transverse orientation through the brain and parallel to the genu and splenium of the corpus callosum in a central slab as indicated in Figure 1a. Imaging of wave data was based on the principle of fractional motion encoding by exploding the broad-band encoding characteristics of a four-period sinusoidal motion-encoding gradient of 60 Hz center frequency (Fig. 1b) [11]. The motion-encoding gradient (MEG) in the through-plane direction was incorporated in a single-shot spin echo echo-planar imaging sequence. Further MRE data acquisition parameters are: time of image sample repetition (TR), 3.0 sec; time to echo (TE), 148 ms; pixel bandwidth, 1.5 kHz; in-plane resolution, 1.5 mm × 1.5 mm; slice thickness, 6 mm; matrix size, 128×128; MEG strength, 35 mT/m. A custom-made head cradle was used for multifrequency head stimulation as described in [2], [12]. The cradle was vibrated by a superposition of four harmonic frequencies of *f* = 25, 37.5, 50, and 62.5 Hz with an input waveform as shown in [11]. Acquisition was repeated 64 times for each image slice with an alternating sign of motion sensitization and an increasing delay between start of vibration and motion encoding. As a result, 32 time-resolved phase-difference wave images were obtained in each image slice.

### Data processing

**Figure 2:**
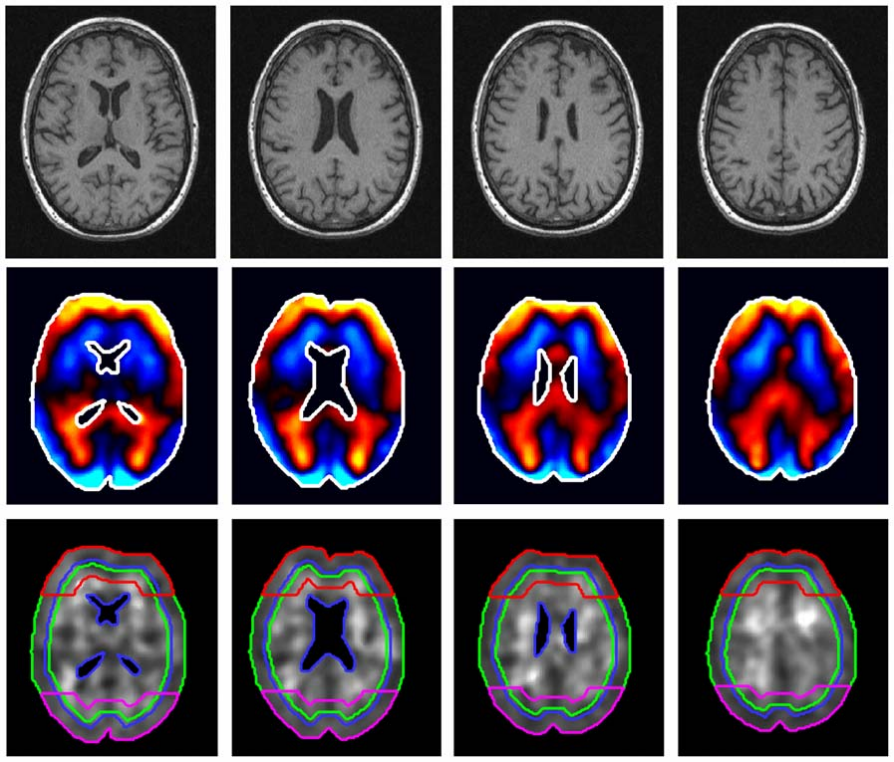
Four image slices from T1-weighted volume MRI data compliant with MRE slice positions (upper row). Color-coded MRE wave data of 50 Hz vibrations. Blue colors scale vibrations towards the reader, while red to yellow colors scale motion beneath the image plane. The maximum tissue deflection is approximately 80* µ*m (mid row). Real-part modulus images corresponding to 50 Hz vibration frequency with specific regions of interest (ROIs) investigated in this study. Green lines: *ROI*_full_, blue lines: *ROI*_inner_, red lines: *ROI*_frontal_, magenta lines: *ROI*_posterior_, outer green lines excluding ventricles: *ROI*_full_ (bottom line).

For each image slice, 32 phase–difference wave images were Fourier-transformed in time for calculating four complex wave images *U*(***x*** *,f*) with *f* = 25, 37.5, 50, and 62.5 Hz. In Figure 2, second row, real part wave images *U*(***x*** *,f*) for *f* = 50 Hz are shown in all four image slices. For comparison, brain biomechanical properties are reported based on the complex shear modulus, G*(***x***,*f*), in the parenchyma excluding the ventricles. Using a locally constant assumption for the complex modulus, G*(***x***,*f*), the relationshipG*(***x***,*f*) = −(2*πf*)^2^ *ρU*(***x*** *,f*)/Δ*U*(***x***,*f*),where *ρ* = 1000 kg/m^3^ is the tissue density and Δ is the 2D-Laplacian, was used to calculate G*(***x***,*f*) in all image planes at each vibration frequency. The global 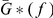 was calculated by averaging G*(***x***,*f*) over all parenchymal spatial points in all planes. According to the springpot model, 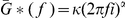, where 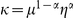. *κ*, *α* are frequency-independent fit variables of 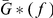. The parameter *µ* is the global shear elasticity, *η* is the viscous damping, and *α* is a measure of the elastic lossy relation. For example, *α* = 0 corresponds to lossless elastic behavior with shear elasticity, *µ* and *α* = 1 to lossy viscous damping with viscosity, *η*. The global storage modulus, 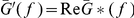, and global loss modulus, 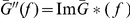, are tabulated. The parameters *κ* and *α* were determined by a least square fit over frequency of the tabulated global 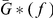 using the springpot model. We present tables of 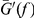, 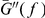, *α*, and *µ*, where for the latter tabulation we assume *η* = 3.7 Pa. This value of *η* was previously determined as an approximated value of viscosity in human brain tissue [2]. As there are no other frequency-independent viscosity values of in vivo human brain in literature we propose this value for translating *κ* to elasticity. This scaling of *κ* from a dimension that depends on *α* (Pa·s*^α^*) to an elasticity improves the comparability of multifrequency MRE results to other elastography studies without changing the significance of the determined mechanical constants.

For illustration *G*' (***x***,*f*) for *f* = 50 Hz is shown in the bottom row of Figure 2. Furthermore, the regions of interest (*ROI*s) we considered are drawn. The main region is *ROI*_full_, which includes the entire brain parenchyma visible in the image slice and is thus associated with ‘global’ mechanical parameters 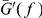, 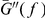, *µ*_full_ and *α*_full_. We also considered four areas located in the cortical, inner, posterior, and frontal regions of the brain as outlined by green (*ROI*_cortex_), blue (*ROI*_inner_), magenta (*ROI*_posterior_), and red lines (*ROI*_frontal_) superimposed on the grayscale images in Figure 2. *ROI*_cortex_ was automatically generated by accounting for a ten-pixel wide ring in the periphery of *ROI*_full_. Inversely, *ROI*_inner_ was derived by eroding eight pixels from the outer edge of *ROI*_full_. *ROI*_frontal_ and *ROI*_posterior_ depict the upper and bottom quarter of a 16-pixel wide ring in the periphery of *ROI*_full_, respectively. The frequency-independent constitutive parameters *µ* and *α* are tabulated for these subregions.

Normalized volumes of the total brain, gray matter (GM), white matter (WM), and brain parenchyma fraction (BPF) were calculated using a method for total brain volume measurement (SIENAX software) with the default BET options (Brain Extraction Tool; part of FSL4.0 Software Library; www.fmrib.ox.ac.uk/fsl).

### Statistical analysis

Results are expressed as arithmetic mean ± standard deviation. Correlations between age, BPF, and total brain volume on the one hand and parameters of viscoelastic properties *µ* and *α* on the other were calculated by Spearman's correlation coefficient. To investigate the influence of age, BPF, and total brain volume (independent variables) on elasticity parameter *µ* (dependent variable), a multivariate linear regression analysis was performed. A two-tailed p-value < 0.05 was considered statistically significant. All tests were performed as constituting exploratory data analyses, such that no adjustments for multiple testing had to be made. All calculations were performed with SPSS Version 18 (SPSS, Inc., Chicago, IL, USA).

## Results

**Figure 3:**
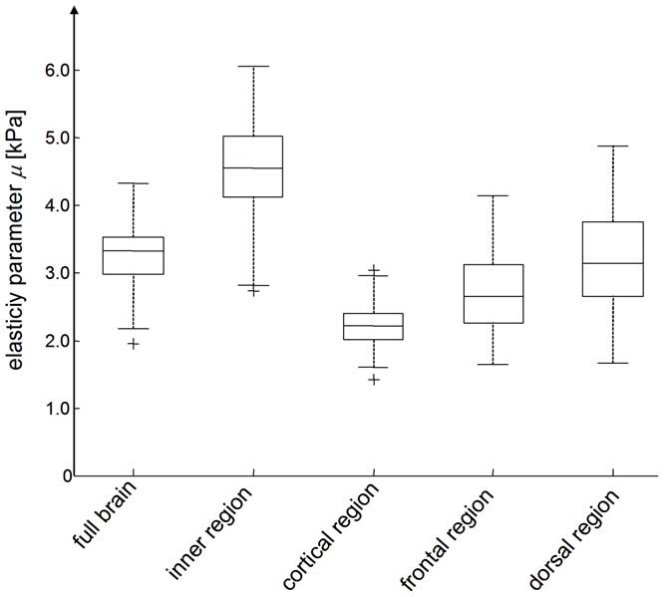
Regional variation in the shear modulus of in vivo brain. All differences between the regions are statistically significant (p<0.001). The boxplot depicts the lower and upper quartiles as well as the median. Full data range (without outliers) is presented by whiskers. Crosses depict outliers.

**Figure 4:**
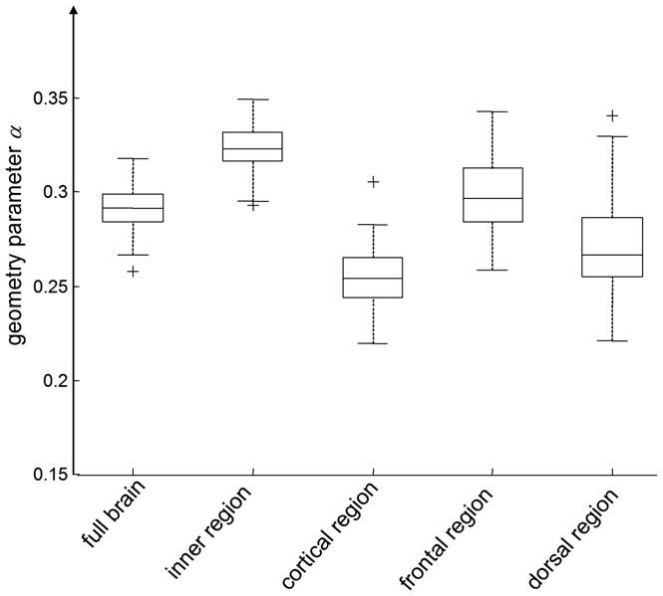
Regional variation in the parameter *α* representing the slope of the modulus dispersion 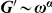 and 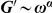 according to the springpot model. As *α* is sensitive to the microstructure geometry of biological tissue it is named ‘geometry’ parameter. Similar to *µ* (Figure 3), all regional differences are statistically significant (p<0.001).

Mean total brain volume and BPF of all subjects were 1.64±0.10 litre and 0.976±0.010, respectively. No significant sex difference was observed in any of the tabulated volume-related parameters (Table 1). Similarly, no significant sex differences in the complex modulus data given in Table 1 were deducible from our cohort. The dispersion of 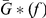
translated to frequency-independent viscoelastic constants *µ* and *α* showed significant (p always < 0.001) variation between regions (Figures 3 and 4). The highest shear modulus was found with *µ*_inner_  =  4.45±0.81 kPa, compared to which *µ*_cortex_  =  2.22±0.35 kPa was reduced by a factor of two. Correspondingly, *α* was highest within the inner region of the brain (*α*_inner_  =  0.323±0.012) and lowest in the cortical area (*α*_cortex_  =  0.255±0.016). All descriptive values of volumes, complex moduli, and springpot-related viscoelastic constants are summarized in Table 1.

**Table 1:** Description of volume data and viscoelasticity parameters.

	All subjects	Men	Women
*Brain volume*			
Volume^a^	1.64 (0.10)	1.64 (0.10)	1.63 (0.09)
Volume WM^a^	0.86 (0.06)	0.87 (0.06)	0.85 (0.06)
Volume GM^a^	0.78 (0.06)	0.78 (0.06)	0.78 (0.06)
BPF	0.976 (0.010)	0.977 (0.008)	0.975 (0.011)
*Storage and loss modulus (full brain)*			
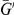 (25 Hz)^b^	1.64 (0.17)	1.66 (0.19)	1.61 (0.15)
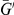 (37.5 Hz) b	1.98 (0.23)	2.01 (0.24)	1.96 (0.23)
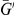 (50 Hz) b	2.12 (0.25)	2.14 (0.27)	2.10 (0.23)
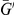 (62.5 Hz) b	2.58 (0.27)	2.64 (0.29)	2.52 (0.24)
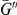 (25 Hz) b	0.80 (0.12)	0.80 (0.13)	0.79 (0.12)
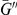 (37.5 Hz) b	0.91 (0.12)	0.94 (0.13)	0.88 (0.11)
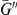 (50 Hz) b	1.08 (0.16)	1.09 (0.17)	1.07 (0.15)
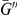 (62.5 Hz) b	1.31 (0.18)	1.36 (0.18)	1.27 (0.17)
*Springpot parameter µ [kPa]*			
*µ*_full_ b	3.25 (0.52)	3.32 (0.57)	3.19 (0.46)
*µ*_inner_ b	4.45 (0.74)	4.50 (0.81)	4.41 (0.69)
*µ*_cortex_ b	2.22 (0.35)	2.28 (0.40)	2.17 (0.29)
*µ*_frontal_ b	3.20 (0.73)	3.47 (0.75)	2.96 (0.63)
*µ*_posterior_ b	2.70 (0.57)	2.66 (0.59)	2.74 (0.55)
*Springpot parameter α*			
*α*_full_	0.291 (0.012)	0.292 (0.012)	0.290 (0.012)
*α*_inner_	0.323 (0.012)	0.323 (0.012)	0.323 (0.013)
*α*_cortex_	0.255 (0.016)	0.256 (0.015)	0.253 (0.016)
*α*_frontal_	0.269 (0.023)	0.273 (0.025)	0.266 (0.021)
*α*_posterior_	0.298 (0.020)	0.300 (0.020)	0.296 (0.020)

The standard deviations (SD) are given in brackets.

a dm^3^,

b kPa.

**Figure 5:**
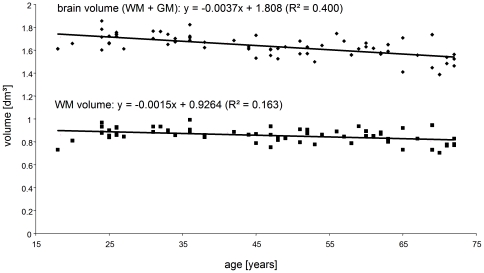
Decrease in total brain volume and WM volume with age represented by linear regression of MRI volume data.

**Figure 6:**
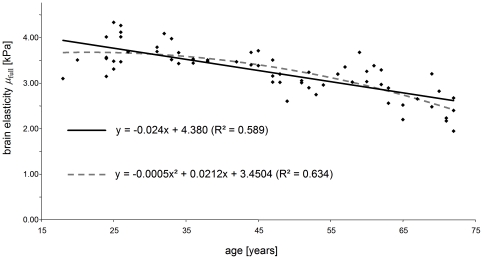
Brain shear elasticity modulus averaged over the entire parenchyma visible in four image slices of all volunteers. Linear and quadratic regression is shown to indicate the order of softening of brain tissue with years of age.

Figure 5 shows the decrease in total brain volume and white matter volume with years of age. All parameters of brain volume (total brain volume, WM, GM, BPF) showed a strong negative correlation with age (Table 2). Total brain volume decreased by 0.23% per year (R-square  =  0.4), whereas the relative annual change in BPF (−0.04%,) was much weaker but correlated better with the linear model (R-square  =  0.42). All complex moduli linearly decreased with age on the order of 0.5% to 0.6% per year with squared correlation coefficients ranging from 0.37 to 0.55. The loss of shear elasticity in the entire brain (*µ*_full_) was even more pronounced with an annual rate of −0.75% (R-square  =  0.59). Figure 6 displays linear and quadratic regressions of *µ*_full_. Compared to linear regression, the correlation of data with the quadratic fit is slightly better (R-square  =  0.63), indicating that there is a maximum of human brain shear modulus during adolescence. The age-related decrease in brain elasticity was most prominent in the interior region of the brain and in the frontal lobe, which is revealed by relative annual changes in *µ*_inner_ and *µ*_frontal_ of −0.80% (R-square  =  0.61) and −0.92% (R-square  =  0.43), respectively. In contrast, *α*_inner_ and *α*_frontal_ were constant or only weakly changing as revealed by annual rates of −0.01% and −0.18% and R-square values of 0.00 and 0.12. The age dependency of all investigated volume- and elasticity-based parameters is given in Table 2. Moreover, Table 2 summarizes the change in *ROI* areas for providing information about possible influences of boundary effects on MRE viscoelasticity data. A very weak interaction of *ROI* area with age was observable. Rates of change and correlation coefficients were similar to those encountered for *α*. The dependency of *µ* and *α* on total brain volume and BPF was investigated by multivariate linear regression. Both *µ* and *α* correlated positively with BPF and total brain volume; however, total brain volume (standardized beta 0.313, p = 0.003) was an independent predictor of *µ* besides age (standardized beta −0.497, p<0.001), while BPF was not (standardized beta 0.110, p = 0.296). *µ* and *α* linearly increased with brain volume by 3.79 kPa/litre (R-square  =  0.488) and 0.05 1/litre (R-square  =  0.178), respectively.

**Table 2:** Age dependencies of volume data and viscoelasticity parameters.

Parameter *X*	Annual change in *X*	Annual change in X [%]	R-square	P
*Brain volume*				
Total volume^a^	−3.72	−0.23	0.40	<0.001
Volume WM^a^	−1.52	−0.18	0.16	0.001
Volume GM^a^	−2.20	−0.28	0.37	<0.001
BPF	−3.89×10^−4^	−0.04	0.42	<0.001
*Storage and loss moduli*				
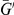 (25 Hz)^b^	−7.71	−0.47	0.53	<0.001
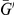 (37.5 Hz)^b^	−10.60	−0.53	0.55	<0.001
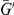 (50 Hz)^b^	−10.64	−0.50	0.48	<0.001
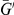 (62.5 Hz)^b^	−11.98	−0.46	0.52	<0.001
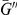 (25 Hz)^b^	−4.61	−0.58	0.37	<0.001
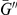 (37.5 Hz)^b^	−5.04	−0.55	0.44	<0.001
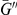 (50 Hz)^b^	−7.09	−0.65	0.52	<0.001
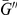 (62.5 Hz)^b^	−7.28	−0.56	0.42	<0.001
*Springpot parameter µ*				
*µ*_full_ b	−24.52	−0.75	0.59	<0.001
*µ*_inner_ b	−35.80	−0.80	0.61	<0.001
*µ*_cortex_ b	−13.93	−0.63	0.42	<0.001
*µ*_frontal_ b	−29.36	−0.92	0.43	<0.001
*µ*_posterior_ b	−12.70	−0.47	0.13	0.003
*Springpot parameter α*				
*α*_full_	−0.00032	−0.11	0.19	<0.001
*α*_inner_	−0.00003	−0.01	0.00	0.784
*α*_cortex_	−0.00025	−0.10	0.07	0.036
*α*_frontal_	−0.00050	−0.18	0.12	0.004
*α*_posterior_	−0.00035	−0.12	0.08	0.020
*MRE ROI*				
*ROI*_full_ c	−24.7	−0.16	0.08	0.023
*ROI*_inner_ c	−19.0	−0.18	0.06	0.039
*ROI*_cortex_ c	−5.7	−0.09	0.09	0.014
*ROI*_frontal_ c	−2.9	−0.10	0.06	0.043
*ROI*_posterior_ c	−1.5	−0.05	0.02	0.319

The annual change is the slope of the regression line with dimensions

a cm^3^/year,

b Pa/year and

c mm^2^/year.

## Discussion

This study represents the first systematic investigation of individual brain geometry and in vivo viscoelastic constants measured by MRE. Brain geometry was quantified using established 3D-MRI and automatic threshold-based image segmentation. We observed a loss of 3.7 cm^3^/year of brain tissue (GM+WM), which is in agreement with previous studies [17], [18], [19]. The decrease in brain viscoelasticity with age was shown in [2] but without taking brain atrophy or regional variations in mechanical parameters into account. This previous study reports mean shear modulus values of total brain of *µ* = 1.94 kPa, which is lower than that observed in the current study. This difference is most presumably due to systematically different slice positions. As mentioned in the Methods section, transverse image slices were aligned through the center of the lateral ventricles other than in [2], [11], [12], [13], where a more peripheral slab of the brain through the upper part or slightly above the ventricles was selected. A higher proportion of sulci in that area might cause increased wave scattering, which reduces apparent wavelengths analyzed by inversion algorithms [20]. The central transverse slab for 2D MRE as used in our current study seems to reproduce elasticity values encountered in 3D MRE [9]. At any rate, the apparent sensitivity of MRE to slice positioning motivates studying geometry–elasticity interactions in cerebral MRE. The results presented here allow us to draw conclusions with respect to i) region-specific *µ* and *α*-values, ii) regional effects of aging on *µ*, and iii) the interaction of brain atrophy and elasticity decrease with age.

### Regional differences in brain viscoelasticity

In literature a large variety of shear modulus values of brain tissue can be encountered as well as different statements about the relative difference in *µ* between white and gray matter [8], [9]. Although the order of our modulus data falls in the range of [9] we observed a lower shear modulus in *ROI*_cortex_ than in *ROI*_inner_, which seems to support observations of [8] about a higher stiffness in WM than in GM. However, it is important to note that our sub-*ROI*s are not related to anatomical structures but to the boundary of the area of brain parenchyma visible in the image slice. *µ*_cortex_ and *α*_cortex_ are rather incidentally correlated with the anatomical subregion of cortical GM, whose peripheral position is inherently susceptible to boundary effects in parameter recovery by discrete wave inversion. Therefore, only limited conclusions about the difference between WM and GM viscoelasticity can be made. The fact that *µ*_cortex_ and *µ*_inner_ differed by more than a factor of two strongly indicates that WM has a higher stiffness than GM. This observation corresponds to the theory that the mechanical matrix of the brain is established by soft-elastic neuronal fibers embedded in even softer glial cells [21]. A higher amount of neuronal fiber tracks would then result in a higher stiffness, particularly in direction of the fiber tracks. However, our study does not account for anisotropic viscoelastic properties of brain matter – a field of research which clearly needs further work. Our interpretation that the neuronal fiber network determines *µ*_inner_ is supported by the higher values of *α*_inner_ compared to *α*_cortex_: According to the theory of the dynamics of G* in generalized Gaussian structures, *α* increases with the degree of vibrational freedoms or, in other words, an increasing (fractal) dimensionality of the mechanical network yields a higher power *α* for 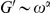 [22], [23].

### Regional effects of aging on brain viscoelasticity

It is known from volumetric MRI that age-related atrophy affects brain regions at different rates. In elderly adults WM atrophy exceeds GM loss and atrophy in the frontal lobe is higher than in the occipital lobe [18]. In contrast, the change rates of *µ*_inner_ and *µ*_cortex_ given in Table 2 suggest a faster decline of GM than WM. However, it is known that GM volume steadily shrinks from early adolescence, while WM volume increases up to the age range of 30 to 40 years [17]. Such age effects may invert the relative atrophy between GM and WM when accounting for younger volunteers. The much higher decrease rate of *ROI*_inner_ compared to *ROI*_cortex_ does not reflect enhanced WM atrophy but an increase of the ventricles with age. There is a trend for *ROI*_frontal_ to decrease slightly with age, whereas *ROI*_posterior_ is not significantly varying. In contrast, *µ*_frontal_ changes with more than −0.92% per year, which is twice the rate of *µ*_posterior_. Since *ROI*_frontal_ and *ROI*_posterior_ are not significantly different, we conclude that the relatively strong age effect on *µ*_frontal_ is mainly due to tissue-intrinsic structure alteration. Taking additionally *α* into account (which is sensitive to the network topology) one can speculate that the geometry of the mechanical tissue matrix is conserved during aging, while the rigidity of structure-building elements such as neurons decreases. Volume seems less influenced by this process since frontal lobe atrophy is on the order of only 0.3% per year [18]. However, a closer look at gyral anatomy by high-resolution anatomical MRI reveals a more rapid atrophy in frontal sulci as compared to occipital sulci [24] which correlates to our observations on *µ*_frontal_ and *µ*_posterior_. Combining these findings, two conclusions can be drawn: On one hand age-related changes in gyral anatomy may have influenced our MRE measures. On the other hand, the degradation of brain tissue and the resulting loss in elasticity may provide the underpinning mechanism for the morphological changes observable by MRI volumetry.

### Interaction of global brain atrophy and viscoelastic parameters

The significant statistical interaction between volume and *µ* as observed in this study does not necessarily imply causal correlation even though both metrics may have one and the same cause. Viscoelastic parameters of biological tissue are related to the connectivity and adhesion of cells and tissue building blocks, which most likely change with age. Yet, since mechanical parameter recovery involves the analysis of strain, i.e., a geometrical quantity, the geometry of brain influences our modulus values. The relative rate of change of total brain volume is about 1/3 that of *µ*. We thus conclude that the maximum influence of atrophy on brain MRE does not exceed 33%.

### Limitations of the study

2D MRE with a slice thickness of 6 mm limits a voxel-based comparison of MRE to MRI volumetry. We have therefore neither normalized MRE data as done in volumetry nor registered MRE to volume data. In volumetry, normalization increased the statistical significance in the observed brain atrophy from R^2^ = 0.19 (non-normalized brain volume) to R^2^ = 0.4 (normalized brain volume) as seen in Figure 5. Aside this improved volume-age correlation, relative and absolute annual rates of atrophy remained widely unchanged. Although 3D MRE including full brain coverage will be mandatory in future studies of volume effects on brain viscoelasticity, the major conclusions of this study about the impact of aging to brain viscoelasticity are attainable by 2D MRE. In general, the high sensitivity of brain MRE to age-related physiological processes motivates further development of the technique towards a clinical modality capable of quantifying widespread neuronal tissue degradation not detectable by other neuroradiological techniques. Brain MRE may particularly benefit from technical advances in parallel imaging [25], anisotropic parameter reconstruction [26] and poroelasticity imaging [27].

In summary, we have demonstrated the high sensitivity of MRE-derived viscoelastic parameters of human brain to physiological aging. The highest rate of change was observed in the frontal area of the brain, which undergoes steady softening with 0.9% per year. In contrast, the slope of the complex modulus dispersion remained widely constant throughout the brain, which is attributed to an unaffected geometrical alignment of mechanically relevant structure elements in brain tissue. Volume-related parameters such as total brain volume, WM and GM volume, and BPF were less sensitive to aging, which demonstrates that viscoelastic parameters are directly related to cerebral tissue structure changing over the lifespan.
